# Long-Term Study of Heart Rate Variability Responses to Changes in the Solar and Geomagnetic Environment

**DOI:** 10.1038/s41598-018-20932-x

**Published:** 2018-02-08

**Authors:** Abdullah Alabdulgader, Rollin McCraty, Michael Atkinson, York Dobyns, Alfonsas Vainoras, Minvydas Ragulskis, Viktor Stolc

**Affiliations:** 1 Prince Sultan Cardiac Center, Alhasa, Saudi Arabia; 2 HeartMath Institute, Boulder Creek, CA USA; 3 Lithuanian University of Health Sciences, Kaunas, Lithuania; 4 Kaunas University of Technology, Kaunas, Lithuania; 5 NASA Ames Research Center, Moffett Field, California, CA USA

## Abstract

This long-term study examined relationships between solar and magnetic factors and the time course and lags of autonomic nervous system (ANS) responses to changes in solar and geomagnetic activity. Heart rate variability (HRV) was recorded for 72 consecutive hours each week over a five-month period in 16 participants in order to examine ANS responses during normal background environmental periods. HRV measures were correlated with solar and geomagnetic variables using multivariate linear regression analysis with Bonferroni corrections for multiple comparisons after removing circadian influences from both datasets. Overall, the study confirms that daily ANS activity responds to changes in geomagnetic and solar activity during periods of normal undisturbed activity and it is initiated at different times after the changes in the various environmental factors and persist over varying time periods. Increase in solar wind intensity was correlated with increases in heart rate, which we interpret as a biological stress response. Increase in cosmic rays, solar radio flux, and Schumann resonance power was all associated with increased HRV and parasympathetic activity. The findings support the hypothesis that energetic environmental phenomena affect psychophysical processes that can affect people in different ways depending on their sensitivity, health status and capacity for self-regulation.

## Introduction

Many studies have been published describing a broad range of physiological, psychological, and behavioral changes associated with changes or disturbances in geomagnetic activity and solar activity. In some countries, magnetic field disturbances are included in public weather forecast reports. On a larger societal scale, increased rates of violence, crime, social unrest, revolutions and frequency of terrorist attacks have been linked to the solar cycle and the resulting disturbances in the geomagnetic field^1–7^. Increased solar activity has not only been associated with social unrest, it is also associated with the periods of the greatest human flourishing with clear spurts of innovation and creativity in architecture, arts, sciences, and positive social change^8^, as well as with variable human performance in the financial markets^9^. The emission of ultraviolet (UV) and solar radio flux radiations (f10.7) modulates with the solar cycle that repeats every 10.5 to 11 years, and increase during the upward phase of the cycle^10,11^. A number of studies have shown that geomagnetic and solar and influences affect a wide range of human behavioral and health outcomes with the nervous and cardiovascular systems being the most clearly impacted, however, the specifics of the biological mechanisms in animals and humans are not yet completely understood^12–15^.

It appears that sharp or sudden variations in geomagnetic and solar activity as well as geomagnetic storms can act as stressors, which alter regulatory processes such as melatonin/serotonin balance^16–18^, blood pressure, breathing, reproductive, immune, neurological, and cardiac system processes^19–22^. Geomagnetic disturbances are associated with significant increases in hospital admissions for depression, mental disorders, psychiatric admission, suicide attempts, homicides and traffic accidents^23–29^. Disturbed geomagnetic activity can also exacerbate existing diseases and is correlated with significant increases in cardiac arrhythmia, cardiovascular disease, incidence of myocardial infarction related death, alterations in blood flow, increased blood pressure, and epileptic seizures^7,20,30–39^.

Heart rate variability (HRV), which is the measurement of beat-to-beat changes in heart rate, is used as an indicator of autonomic nervous system (ANS) function and dynamics. Use of HRV has substantially increased in recent decades in research and clinical treatment applications^40–43^. HRV can be assessed with various analytical approaches, although the most commonly used are frequency domain (power spectral density) analysis and time domain analysis. In both methods, the time intervals between each successive pair of heartbeats (called inter-beat-intervals or IBIs) is first measured. Power spectral analysis separates the complex HRV waveform into the individual rhythms, each having a different underlying physiological mechanism and osculates within different frequency ranges. The power spectral density values reflect the area under the curve within the specific bandwidth of the spectrum. The interactions between autonomic neural activity, blood pressure, respiration and higher level control centers in the brain produce both short and longer-term rhythms in HRV measurements^40^. Not to be confused with the frequency bands used in radio communications, the North American Society of Pacing and Electrophysiology and European Society of Cardiology Task Force Report on HRV separated heart rhythm fluctuations into three primary frequency bands: high frequency (HF), low frequency (LF), and very low frequency (VLF)^44^. The HF range equates to rhythms with periods that occur between 2.5 and 7 seconds. This band reflects parasympathetic activity primarily related to the respiratory cycle. The LF range equates to rhythms and modulations with periods that occur between 7 and 25 seconds. In ambulatory 24-hour HRV recordings, it has been suggested that the LF band reflects sympathetic activity in addition to baroreceptor activity involved in short-term blood pressure regulation. The LF/HF ratio has been controversially used to assess the balance between sympathetic and parasympathetic activity, especially in short-term resting recordings^45–47^. A number of researchers have challenged this perspective and have persuasively argued that in resting conditions, the LF band only reflects baroreflex activity and not sympathetic activity^48^. The VLF is the power in the rhythms or modulations with periods that occur between 25 and 300 seconds. Although all 24-hour measures of HRV reflecting low HRV are linked with increased risk of adverse outcomes, the VLF band has stronger correlations with all-cause mortality than the other bands^49–52^. Relatively new evidence has suggested that the VLF oscillations are generated by the intrinsic cardiac nervous system within the heart and that the frequency and amplitude of these rhythms are modified by sympathetic efferent activity. Sympathetic activations can increase the VLF power and cross over into the lower region of the LF band when there is a significant emotional stressor or during periods of physical activity^53^. Low VLF power is also associated with arrhythmic death^54^ PTSD^55^, and high inflammation^56,57^ and has been correlated with low levels of testosterone^58^. Total power (TP) is a measure of all the HRV bands combined, and therefore is a measure of the overall HRV from all physiological sources, although it is highly affected by the VLF power.

Multiple studies have demonstrated significant decreases in HRV during magnetic storms indicating a possible mechanism linking geomagnetic activity with increased incidents of coronary disease and myocardial infarction and suggest that the cardiovascular system is a clear target for the impact of geomagnetic distrubances^15,22,30,59–66^. Several studies that analyzed week-long recordings found about 25% reduction in the VLF activity during magnetically disturbed days as compared to quiet days^61–63,67^. Lower activity or power in the VLF rhythm has been most highly correlated with increased risk of death from all causes while the vagally mediated HF rhythm is not as predictive, although lower activity in HF rhythm is associated with decreased capacity to self-regulate thoughts, emotions and behaviors^40,50^. Dimitrova *et al*., found that both HF and LF bands are reduced during geomagnetic storms and that the overall response patterns of participants varied in response to space weather parameters^60^.

A number of studies have also observed an anticipatory reaction that can occur several days before the onset of a magnetic storm with significant alterations in participants’ blood pressure, HRV, heart rate, skin conductance and physiological symptoms^15,60,68–71^. This anticipatory reaction was first observed by Tchizhevsky and other scientists prior to the measurements of X-ray, and gigahertz frequency (f10.7) emissions from the sun. Tchizhevsky suggested some type of unknown radiation produced by the sun was likely responsible^70^. The increased solar radiations that Tchizhevsky referred to and that are associated with coronal mass ejections, only take 8-minutes to travel to the Earth as opposed to the stream of plasma emitted from the sun that travels with the solar wind, which requires up to 3-days to impact the magnetosphere of the Earth resulting in the onset of a magnetic storm. Khabarova has suggested that a possible mechanism for this anticipatory affect may be due to a reorganization of the ionosphere currents from increased solar electromagnetic radiation^70^.

Stoupel *et al*. have examined periods of low levels of geomagnetic disturbance combined with higher levels of cosmic ray activity and found there was a significant rise in emergency calls and overall deaths during these periods, with the most increases in cerebral strokes and sudden cardiac death suggesting that cosmic rays are an important factor affecting human medical events in elder populations^15,72,73^.

In a review of the research literature on health effects of geomagnetic disturbances, Palmer *et al*. observed these “definite conclusions”: (1) Geomagnetic disturbances have a greater effect on humans at higher geomagnetic latitudes. (2) Unusually high values of geomagnetic activity (disturbance) have a negative effect on human cardiovascular health. (3) Unusually low values of geomagnetic activity seem to have a negative effect on human health. (4) Only 10% to 15% of peoples’ health is negatively affected by disturbances in geomagnetic activity and (5) HRV is negatively correlated with disturbances in geomagnetic activity^74^.

Less attention has been paid to the ultralow frequency (ULF) which is generally descried as magnetic activity less than 5 Hz in the geomagnetic literature, on health and physiological function. Field-line resonances are the most common source of ULF wave energy measured on the ground and exhibit the largest wave amplitudes compared to other oscillations that occur in the magnetosphere^75^. The frequency of the field-line resonances is affected by plasma density, the length of the magnetic field-lines, and the field strength. Oscillations with frequencies below 1 Hertz are classified according to their frequency and waveform and quasi-sinusoidal oscillations are classified as “Pc” (pulsation continuous). Waves with irregular shapes are classified as “Pi” (pulsation irregular). Standing wave field-line resonances are typically classified as Pc3 to Pc5 waves corresponding to frequencies between 1 mHz and 100 mHz. Pc1 and 2 oscillations are classified as traveling waves, which can have frequencies up to 5 hertz, and are typically excited by geomagnetic substorms^76^. Studies have found that increases in field-line resonances can affect the cardiovascular system, which may be because the Pc waves are in a comparable range with those of the autonomic nervous and cardiovascular systems^77^. Khabarova and Dimitrova demonstrated that the magnetic waves in the 2–10 mHz region had the higher correlations with increased blood pressure than geomagnetic indices^69^. In addition, Zenchenko et.al found there was a significant degree of synchronization between HRV rhythms and the osculation’s in geomagnetic field in the frequency range between 0.5 to 3.0 mHz in two-thirds of their experiments of over periods of 4 to 30 minutes^78^. It has also been demonstrated that ANS activity not only reacts to shifts in geomagnetic and solar activity, it can also synchronize with rhythms in the time-varying magnetic fields related to the Schumann resonances (SR) and geomagnetic field-line resonances^15,79^.

In the 1950s Winfried Otto Schumann and Herbert Koenig first measured frequencies that were similar to a mathematical model that predicted a magnetic wave resonance between the Earth and ionosphere^80^. The first Schumann resonance frequency is 7.83 hertz (Hz), with a (day/night) variation of around ±0.5 Hz. The higher frequencies are ~14, 20, 26, 33, 39 and 45 Hz, all of which closely overlay with alpha (8–12 Hz), beta (12–30 Hz) and gamma (30–100 Hz) brain waves. The similarity of the electrical components of the time-varying voltages generated by the brain (EEG) with the SRs was recognized early on, and the ability for the EEG rhythm to synchnorize with SR activity was observed by Koenig^81^.

Pobachenko *et al.*^82^ examined the SR and the EEGs in a group of participants over six-weeks and found that during the daily cycle, changes in the EEG were similar to variations in the SR. The highest correlations between the SRs and brain rhythms occurred when the magnetic activity was increased. Persinger *et al*. have also studied SR and EEG activity the in real-time and have shown that many of the SR frequencies can be observed in the power spectrums of most human brain activity^83,84^. They have also shown that the spectral profiles within the EEG activity displayed recurrent transient segments of real-time coherence (synchronization) with the first three resonant frequencies of the SRs (7–8 Hz, 13–14 Hz, and 19–20 Hz). This suggests that under certain conditions, variables affecting the Schumann parameters (such as solar wind) may affect brain activity, such as modifications of perception and dream-related memory consolidation^84^. Altered EEG rhythms in response to changing magnetic fields have also been observed by Belov *et al*., with low frequency magnetic oscillations (around 3 Hz) having a sedative effect^85^.

In the study reported here, the potential correlations between solar and magnetic factors and the time course or lags in autonomic nervous system responses to changes in solar and geomagnetic activity were examined by collecting HRV data for seventy-two consecutive hours each week over a five-month period using ambulatory HRV recorders. The intent was to examine the group’s ANS responses, as reflected in HRV, to changes in solar and geomagnetic background activity over an extended period. The maximum Ap Index level for each day during the study period is summarized in Table 1 and provides an overview of the general level of geomagnetic activity during the study period (April 1 – August 31, 2012).

**Table 1:** Summary of Study Period Geomagnetic Activity.

Category	Ap index range	Days, n
Quiet	<8	41
Unsettled	8–16	48
Active	16–30	35
Minor storm	30–50	17
Major storm	50–100	9
Severe storm	>100	3

## Methods and Procedures

### Participants

Eighteen females, all healthy employees of the Prince Sultan Cardiac Center in Hofuf, Saudi Arabia (8 nursing staff, 6 housekeeping and, 4 from the research department) volunteered to participate in this study. The average age was 32 ± 8 years, ranging from 24–49 years. Inclusion criteria were no known physical or mental health disorders and were fulltime employees. Exclusion criterion was a known health disorder, or taking any medications known to affect autonomic function. All participants signed informed consent and were free to withdraw from the study at any time. Two participants experienced uncomfortable irritation at the ECG electrode sites and dropped out of the study. The research was performed in accordance with all relevant guidelines and met all applicable regulations for the ethics of experimentation and was approved by the Ministry of Health Research Centers ethical decision party (R.C. 100/25).

### Data Collection

All participants underwent weekly 24–72 hour ambulatory HRV recordings with Bodyguard HRV recorders (Firstbeat Technologies Ltd, Finland). The recorder samples the ECG at 1000 Hz and calculates the inter-beat-interval (IBI), which is the time in milliseconds between consecutive heartbeats. IBI data was stored locally in the device memory and uploaded loaded to an FTP site at the end of each week. Participant recordings were generally 72-hours in length and were scheduled once a week over a 5-month period between April and the end of August 2012. A total of 960 twenty-four hour long HRV recordings were obtained.

### Measures

#### Heart Rate Variability

The HRV measures used in this study were the IBI, Total Power, LF, and HF power, and the LF/HF ratio. All HRV recordings were downloaded from the FTP site to a PC workstation and analyzed using DADiSP 6.5. Inter-beat-intervals greater or less than 30% of the mean of the previous 4 intervals were considered artifacts and removed from the analysis record. Following an automated editing procedure, all recordings were manually reviewed by an experienced technician and, if needed, corrected. Daily recordings were processed in consecutive 5 minute segments in accordance with the standards established by the HRV Task Force^86^. Any 5 minute segment with >10% of the IBIs either missing or removed in editing were excluded from analysis. Results of the 5-minute segments were averaged into hourly values to match the resolution of the environmental data sets. Local HRV data timestamps were converted to UTC and synchronized with the environmental data sets.

#### Environmental Measures

**Figure 1:**
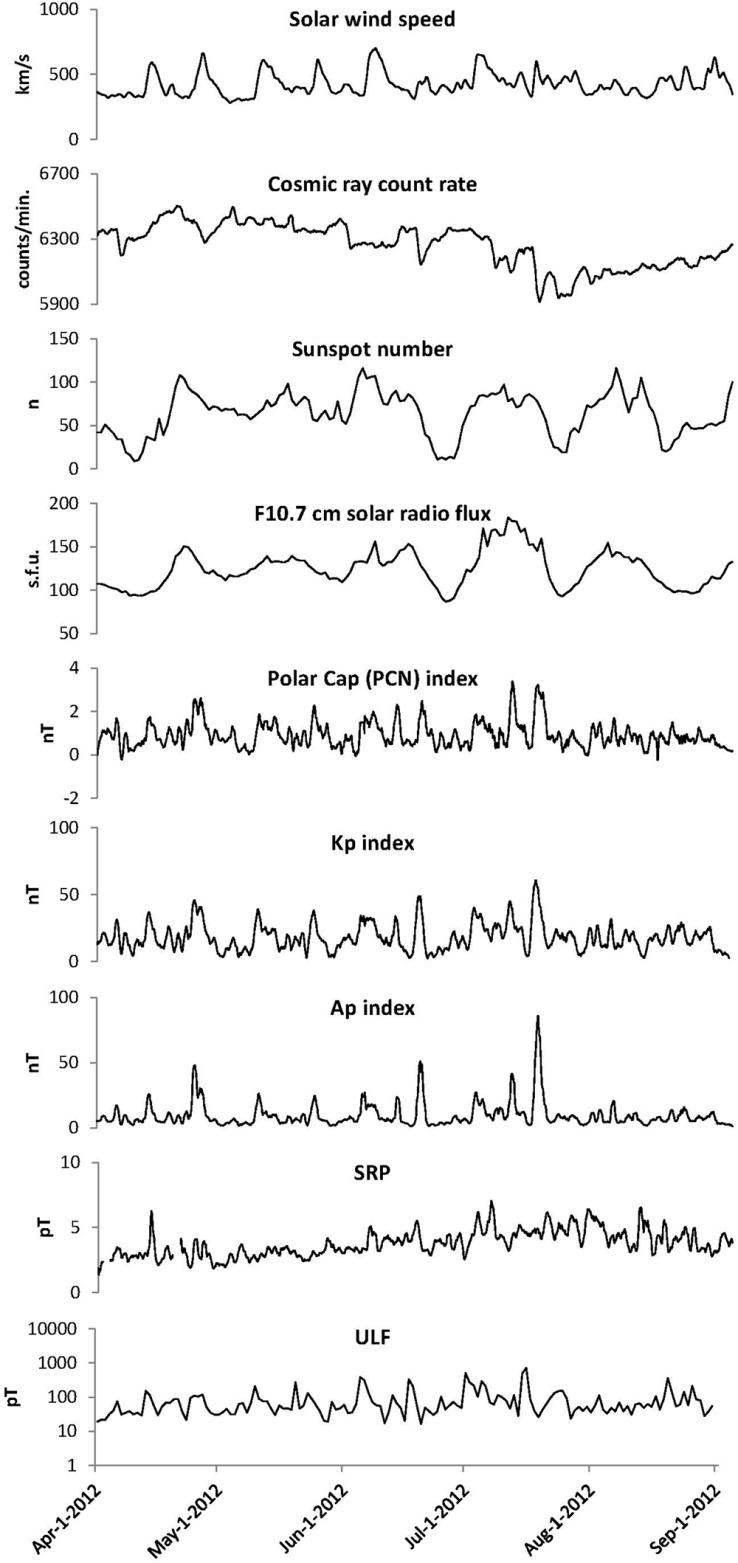
Environmental data activity across the study period. There was a large increase in the Kp and Ap indexes that occurred on July 14^th^, which resulted from a coronal mass ejection that hit the earth’s magnetic field at approximately 1800 UT that day.

Space weather and environmental measures were obtained from three sources, comprising nine measures. The solar wind speed, Kp index, Ap index, number of sunspots, F10.7 index, and the geomagnetic polar cap index (PCN) were downloaded from NASA Goddard Space Flight Center’s Space Physics Data Facility as part of the Omni 2 data set. Cosmic ray counts were downloaded from Finland’s University of Oulu’s Sodankyla Geophysical Observatory’s website. Power in the time varying magnetic field in two frequency bands, Schumann Resonance Power (SRP), 3.5 to 36 Hz and ULF power, 2 mHz to 3.5 Hz were obtained from a recording site located in Boulder Creek, California. Table 2 summarizes frequency band ranges for the HRV and magnetic field measures used in this study. The HeartMath Institute maintains a network of highly sensitive induction coil magnetometers (Zonge ANT-4; sensitivity 10^−12^ T) as part of a special project called the Global Coherence Initiative^87^. Each site includes two magnetometers positioned in the north-south and east-west axis to detect local time varying magnetic field strengths over a relatively wide frequency range (0.001–50 Hz) while maintaining a flat frequency response. The data acquisition infrastructure collects and timestamps all data using GPS time signals before uploading to a common server. Each magnetometer is continuously sampled at a rate of 130 Hz. Figure 1 shows the time domain data for the environmental data activity across the study period.

**Table 2:** Summary of magnetic and HRV frequency ranges used in measurements.

Measurement frequency ranges	
	Hz
Schumann resonance power	3.5–36
Magnetic field ULF power	0.002–3.5
**HRV**	
Total power	0–0.4
Very low frequency	0.003–0.04
Low frequency	0.04–0.15
High frequency	0.15–0.4

### Statistical Analysis

#### Regression Analysis

These data were analyzed by means of multivariate linear regression. Each HRV variable is considered separately as a single dependent variable. In the first analysis stage, each participant’s dataset is handled separately. For each HRV variable and each participant dataset, regression coefficients were computed to minimize the total squared error of a linear model. The assumed model is that

1 $${HRV}={{R}}_{1}{{E}}_{1}+{{R}}_{2}{{E}}_{2}+\mathrm{...}$$

where HRV is the dependent HRV variable, E1, E2, etc., are the independent environment variables, and R1, R2, etc., are the regression coefficients. Before the model results were calculated, hourly observations, which have missing data in any variable, were discarded so that only observations with a complete set of environmental measures were used. This so-called “complete cases” approach is a standard method for dealing with missing data in regressions. In addition, the data were normalized so as to eliminate circadian confounds. A side effect of the circadian normalization is that it forces both dependent and independent variables to have an overall mean of zero over the observation period, so that the regression model can safely ignore “intercept” terms and compute only “slopes”, as implicit in the model described above.

**Figure 2:**
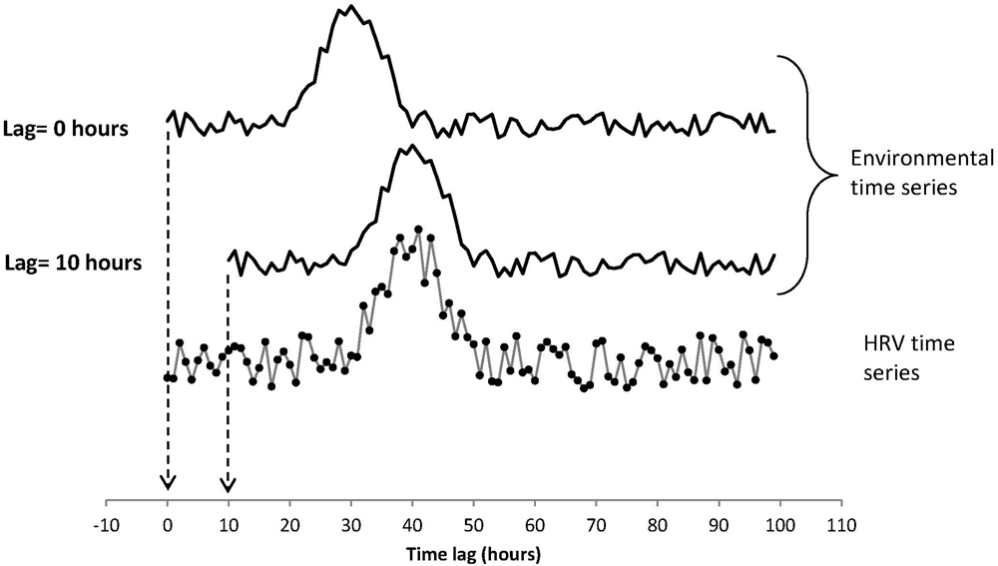
Two data series representing an environmental time series and a HRV time series. The environmental signals position at lag 0 (upper series) is as it occurred relative to the HRV signal. The middle trace shows the same environmental data series after shifting it in time by 10 hours. In this example, correlations between the HRV and environmental data would be higher at lag 10 than at lag 0 indicating a delayed physiological response to the external environmental signal.

In addition to the considerations above, the analysis also evaluated possible time-offset delayed effects, since it is known that HRV measures may show a reaction to a stimulus several hours after the stimulus is applied. The hourly timestamps in both HRV and environment data allow each set of HRV measurements to be linked unambiguously to a set of environment measurements. These can also be used to examine behavior with a lag in response time simply by offsetting the timestamps that are to be matched. Temporal offsets ranging from zero to 40-hours were used, meaning that the calculation described above was actually repeated 41 times (counting the 0-offset model for matched times). Figure 2 illustrates how time-offsets are used to examine lags in response times.

The result of all of these calculations is a multidimensional array of regression coefficients with their associated standard errors, computed by the R functions “lm” and “summary.lm”. There is one regression coefficient (and computed statistical standard error) for each of six HRV variables, as influenced by each of nine environment variables, for each of 16 participants at each of 41 time lags. The next analysis step after computing the models was to compute a cross-participants’ average for the regression coefficient, thereby eliminating the “participants” dimension of the multidimensional array. This average is the mean of the participants’ regression coefficients, individually weighted by their associated standard errors in the usual least-squares formula:

2 $$M=\frac{\sum {x}_{i}/{\sigma }_{i}^{{\rm{2}}}}{\sum {\rm{1}}/{\sigma }_{i}^{{\rm{2}}}}$$

where M is the weighted mean, *x*_*i*_ are the individual observations, and σ_*i*_ are their associated measurement uncertainties. (This weighting is “standard” because it minimizes the total squared error between the mean and the individual observations).

The result of this calculation is that the array of individual participant observations can be replaced by a single value, the weighted mean, with an associated measurement uncertainty.

3 $${\sigma }_{M}^{{\rm{2}}}=\sum 1/{\sigma }_{i}^{{\rm{2}}}.$$

In addition to these two values, the statistical evaluation of the participant-averaged data also computed the Z-score

4 $$Z=M/{\sigma }_{M},$$

the p-value of each Z-score, a chi-squared value expressing the amount of excess variation between participants beyond that expected from the regression uncertainties, and the p-value of this chi-squared. These last few values relate only to inter-operator variability, which is beyond the scope of the current analysis. The Z-score, however, was used extensively in both analysis and visualization, since it is directly related to the statistical significance of a given result and provides a scale-independent measure for comparing the contributions of different environment variables. Z-scores are sometimes referred to as standard normal deviates. Strictly speaking, this assumes that the errors are normally distributed, but since the calculation discussed here is the mean of sixteen independent models computed for the sixteen participants, any departures from normality will be very small regardless of the underlying distribution of observational errors for an individual participant.

Multiple Analysis Corrections: After averaging across the contributions of the participants, one is still left with a multidimensional array of results for nine environment variables, by six HRV measures, by 41 time lags, for a total of 2214 analysis results. As insurance against introducing analysis errors by selecting significant results that appear purely by chance, we applied a Bonferroni correction for multiple analyses to this array of analysis results, or to any subset that we may be examining in more detail. The usual Bonferroni correction is made by multiplying the p-value of the most significant analysis by the number of analyses. Strictly speaking, the proper correction for N analyses, with the strongest result having a p-value of P, is

5 $${P}_{corr}={\rm{1}}-{({\rm{1}}-P)}^{N}.$$

This exact formula expands, however, into

6 $${P}_{corr}=NP-O({P}^{{\rm{2}}}).$$

The simple formula

7 $${P}_{corr}=NP$$

can thus be seen to be conservative, always larger than the actual p-value, with an error that is negligible for P ≪ 1. For the record, the strongest individual result in this full analysis array has a p-value of 1.4 × 10^−32^, which becomes 3.1 × 10^−29^ after Bonferroni correction for 2214 analyses.

#### Lag Analysis

As noted above, the multiple-analyses corrections must include the consideration that 41 different time lags were examined by the same regression methods. When allowing for the fact that the actual relationship between geomagnetic environmental variables and an HRV measure might involve an unknown time lag, the most straightforward way of dealing with the multiple analysis problem is to apply a 41-test Bonferroni correction to the lag results for a single HRV-to-environment regression coefficient. This allows one to draw a valid conclusion for whether that coefficient is displaying a statistically significant value at any of the time lags examined. In graphical visualizations of the lag results, this is often done implicitly by plotting the unmodified series of Z-scores and placing a 5% significance envelope at ±3.24, the two-tailed 5% threshold for the largest of 41 standard normal deviates (1% significance at ±3.67).

The analysis has 54 such coefficients, analyzing six HRV measures by nine independent variables. Some evaluations, such as an overall significance for the entire model, require that a factor of 54 Bonferroni correction be applied to the most significant individual results. Other types of analysis, such as evaluating how many of the regression coefficients are individually significant at some time lag, require no further Bonferroni correction beyond the one applied for the multiplicity of lags.

## Results

Environmental and HRV measure correlation results are provided in Tables 3 and 4. In agreement with previous studies, solar wind speed was highly correlated with Kp (r 0.50, p < 0.01) and Ap (r 0.35, p < 0.01) indexes, ULF power and negatively correlated with cosmic ray counts (r −0.15, p < 0.01)^88–90^. As expected, the solar radio flux (F10.7) was also highly correlated with the number of sunspots (r 0.81, p < 0.01). The Schumann Resonance Power was negatively and highly correlated with cosmic ray counts (r −0.58, p < 0.01). ULF power was positively correlated with solar wind speed (r 0.44, p < 0.01), Kp (r 0.58, p < 0.01), Ap (r 0.61, p < 0.01) and PC(N) (r 0.43, p < 0.01) indexes. The correlations among the HRV variables were as expected and in agreement with other studies with the exception of HF power, where correlations to IBI, Total Power, and VLF power were all higher than seen in individual 24-hour recordings (Table 4)^91^.

**Table 3:** Environmental measure correlations

Environmental measure correlations, circadian rhythm removed										
		1	2	3	4	5	6	7	8	9
1.	Solar wind speed	1	0.50**	0.35**	0.10**	0.17**	0.42**	−0.14**	0.23**	0.44**
2.	Kp index	0.50**	1	0.90**	0.18**	0.30**	0.87**	−0.19**	0.15**	0.58**
3.	Ap index	0.35**	0.90**	1	0.19**	0.30**	0.82**	−0.20**	0.14**	0.61**
4.	Sunspots, n	0.10**	0.18**	0.19**	1	0.81**	0.24**	0.10**	0.11**	0.15**
5.	F10.7 index	0.17**	0.30**	0.30**	0.81**	1	0.35**	−0.05**	0.24**	0.18**
6.	PC(N)	0.42**	0.87**	0.82**	0.24**	0.35**	1	−0.08**	0.09**	0.43**
7.	Cosmic ray, counts	−0.14**	−0.19**	−0.20**	0.10**	−0.05**	−0.08**	1	−0.58**	−0.15**
8.	SRP	0.23**	0.15**	0.14**	0.11**	0.24**	0.09**	−0.58**	1	0.28**
9.	ULF	0.44**	0.58**	0.61**	0.15**	0.18**	0.43**	−0.15**	0.28**	1

*p < 0.05, **p < 0.01.

**Table 4:** HRV measure correlations.

HRV measure correlations, circadian rhythm removed									
		1	2	3	4	5	6	7	8
1.	IBI, ms	1	0.72**	0.89**	0.86**	0.86**	0.68**	0.87**	−0.65**
2.	SDNN, ms	0.72**	1	0.86**	0.83**	0.85**	0.79**	0.86**	−0.47**
3.	ln RMSSD, ms	0.89**	0.86**	1	0.95**	0.94**	0.89**	0.99**	−0.55**
4.	ln TP, ms^2^/Hz	0.86**	0.83**	0.95**	1	0.99**	0.94**	0.92**	−0.34**
5.	ln VLF, ms^2^/Hz	0.86**	0.85**	0.94**	0.99**	1	0.92**	0.90**	−0.35**
6.	ln LF, ms^2^/Hz	0.68**	0.79**	0.89**	0.94**	0.92**	1	0.87**	−0.14**
7.	ln HF, ms^2^/Hz	0.87**	0.86**	0.99**	0.92**	0.90**	0.87**	1	−0.61**
8.	ln LF/HF	−0.65**	−0.47**	−0.55**	−0.34**	−0.35**	−0.14**	−0.61**	1

*p < 0.05, **p < 0.01.

**Figure 3:**
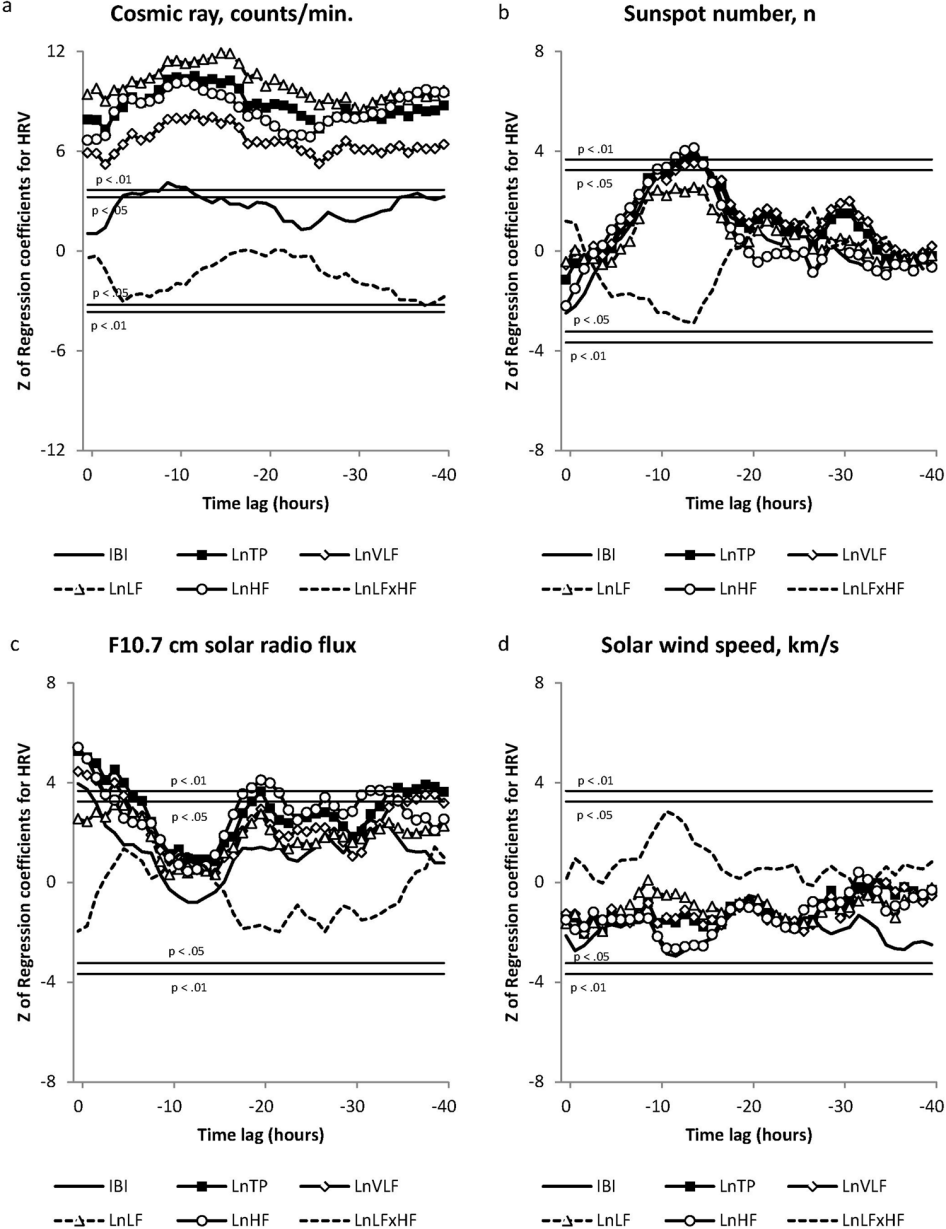
The correlations between HRV variables and changes in measures of solar activity and cosmic rays across the forty-hour analysis time period.

**Figure 4:**
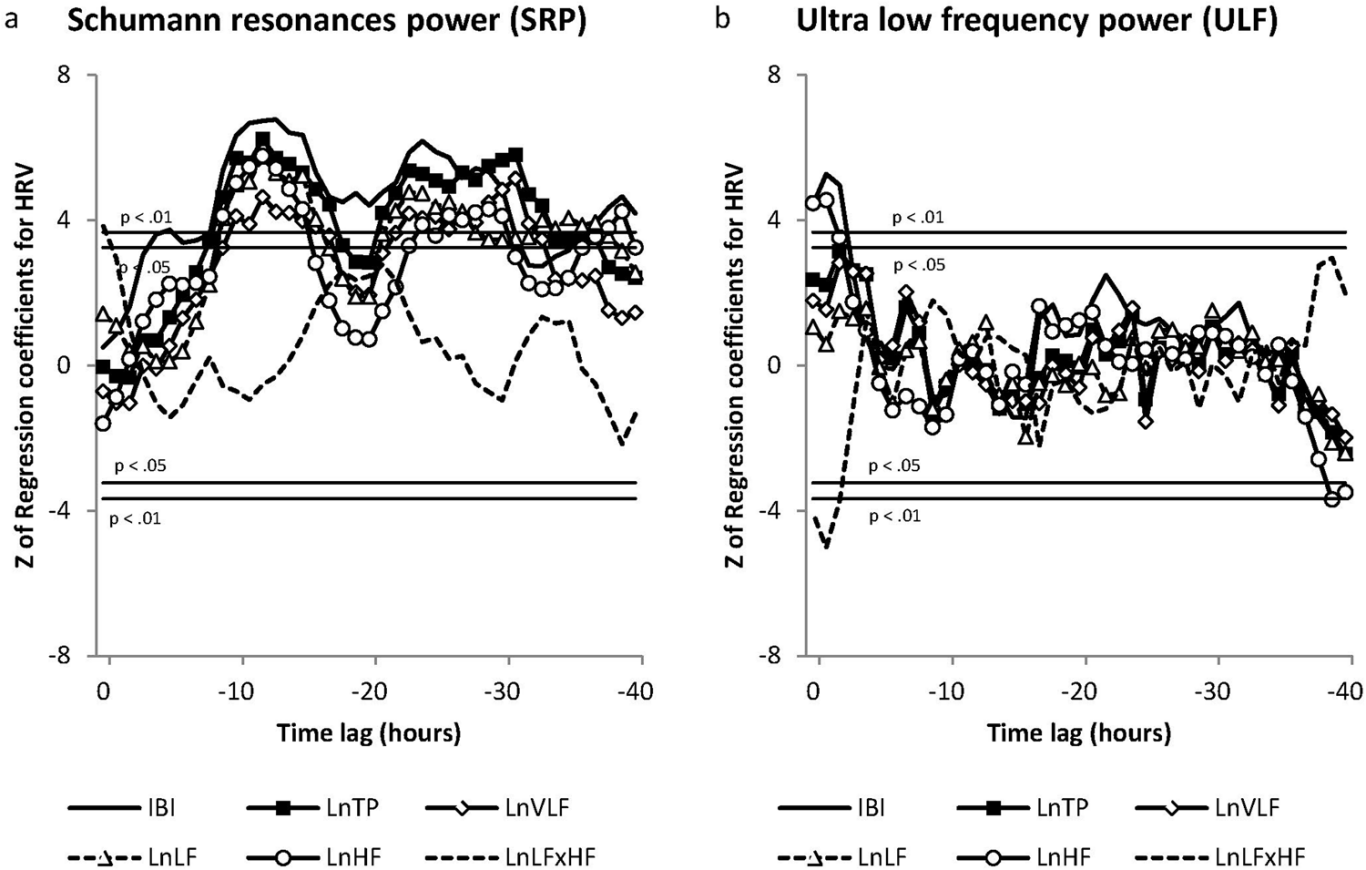
The correlations between HRV variables and changes in Schumann Resonances and ULF power across the forty-hour analysis period.

**Figure 5:**
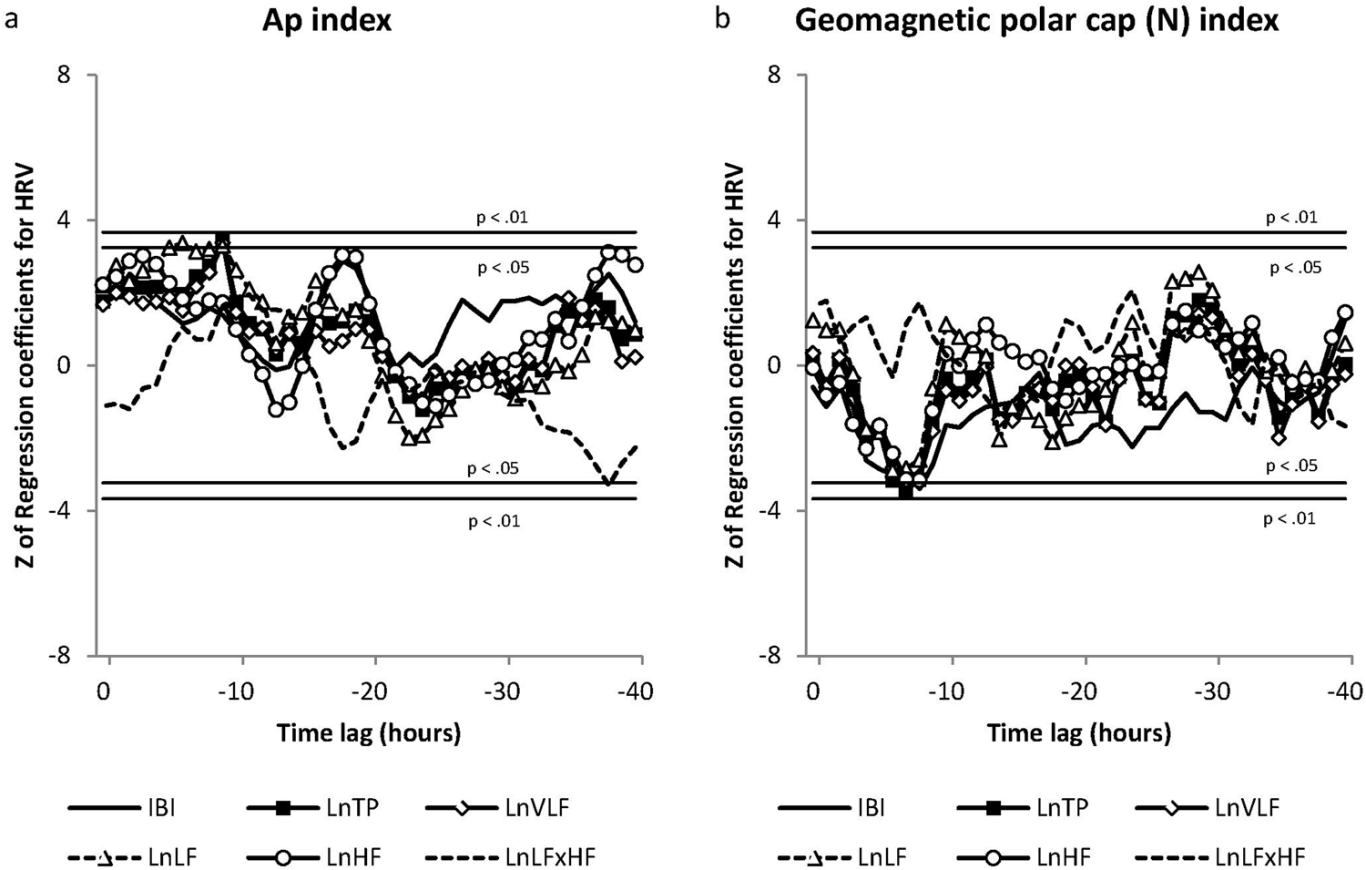
The correlations between HRV variables and changes in measures of magnetic field disturbance across the forty-hour analysis time period.

Results of the multivariate linear regression between the environmental and HRV variables revealed a number of significant findings with different HRV metrics responding at different time lags with changes in environmental factors. Z-scores for regression coefficients are shown in Figs 3, 4 and 5. Detailed Z-score tables were too large for inclusion here and can be found in the supplemental information. There were significant autonomic nervous system responses reflected in the HRV to changes in cosmic ray counts (Fig. 3a), which were strong and consistent. The TP (Z 7.30 to Z 10.49, p < 0.01), VLF (Z 5.20 to Z 8.19, p < 0.01), LF (Z 8.49 to Z 11.88, p < 0.01) and HF (Z 6.63 to Z 10.16, p < 0.01), all quickly and strongly responded, and were positively correlated across the entire 40-hour period. IBIs responded from hour 4 through 12, and again at hours 36, 37 and 40 (Z 3.32, p < 0.01 to Z 4.11, p < 0.01). The negatively correlated LF/HF ratio was significant only at hour 38 (Z −3.33, p < 0.05).

For changes in the number of sunspots (Fig. 3b), IBIs were positively correlated in hour 12 through 15 (Z 3.42, p < 0.05 to Z 3.84, p < 0.01). TP was significant at hour 10, and again during hours 12 to 15 (Z 3.25, p < 0.05 to Z 3.73, p < 0.01). HF was also positively correlated from hour 10 to hour 15 (Z 3.28, p < 0.05 to Z 4.13, p < 0.01). The VLF was nearly the same, but was not significant until the 13^th^ hour and remained significant through hour 15 (Z 3.43 to Z 3.52, p < 0.05). The LF and LF/HF ratio were non-significant.

For solar radio flux (F10.7) (Fig. 3c), there was an immediate, robust positively correlated response in the IBIs, TP, VLF and HF power. The IBI response was significant for the first two hours (Z 3.73 to Z 3.96, p < 0.01). The TP power response remained significant over the first 8-hours, and again during the 19^th^ and 20^th^ hour and from hour 34 to the end of the analysis period (Z 3.25, p < 0.05 to Z 5.26, p < 0.01). The VLF response was significant over the first 6-hours, and at hour 35, 37, 38, and 39 (Z 3.31, p < 0.05 to Z 4.44, p < 0.01), while the HF response was significant during the first 5 hours, the 18^th^ through 22^nd^ hours and the 31^st^ to 35^th^ hours (Z 3.30, p < 0.05 to Z 5.40, p < 0.01). The LF and LF/HF ratio did not reach significance at any point in the analysis period. The solar wind speed was not correlated with any of the HRV variables across the 40-hour period.

Figure 4a shows the correlations for Schumann Resonance Power (SRP) and Fig. 4b ULF Power. There were a number of significant correlations. For the SRP, the IBIs were the first to respond, and became significant in the 4^th^ and remained significant through hour 31 and again at the 36^th^ to 40^th^ hour (Z 3.37, p < 0.05 to Z 6.76, p < 0.01). The Total Power response was significant during hours 8 to 18 and from hours 21 to 37 (Z 3.30, p < 0.05 to Z 6.23, p < 0.01). VLF power was significant from hours 10 to 17 and again at hours 22 through 33 (Z 3.46, p < 0.05 to Z 5.14, p < 0.01). LF power became significant starting at hour 9 and continuing through hour 16 and again between hours 21 and 38 (Z 3.45, p < 0.05 to Z 5.74, p < 0.01). In addition, HF power was positively correlated between hours 9 to 15, hours 23 to 30, and hours 37 to 39 (Z 3.28, p < 0.05 to Z 5.76, p < 0.01). The LF/HF ratio was correlated only during the first hour (Z 3.86, p < 0.01).

In response to changes in ULF power, there was an immediate positively correlated response lasting 3 hours in the IBIs (Z 4.42 to Z 5.26, p < 0.01), and HF power (Z 3.51, p < 0.05 to Z 4.54, p < 0.01). HF power became negatively correlated during the last two hours (Z −3.49, p < 0.05 to Z −3.69, p < 0.01). The LF/HF ratio was negatively correlated during the first three hours (Z −3.72 to –Z 5.02, p < 0.01).

Keeping in mind the relatively few magnetic disturbances that occurred during the study period, there were very few correlations with the indices of geomagnetic disturbances. The Polar Cap activity (PC.N) (Fig. 5b) had a significant negative correlation with TP in the 7^th^ hour (Z-3.42, p < 0.05), and IBI in the 8^th^ hour (Z −339, p < 0.05). There were no significant correlations to the Kp Index. The Ap index (Fig. 5a), was positively correlated with TP, VLF, and LF in the 9^th^ hour (Z 3.42, Z 3.37 and Z 3.30, p < 0.05) respectively, and LF was also significant in the 6^th^ hour (Z 3.37, p < 0.05). The LF/HF ratio was negatively correlated during the 38^th^ hour (Z −3.29, p < 0.05).

## Discussion

Overall, this study confirms that daily ANS activity, as reflected by HRV measures, reacts to changes in geomagnetic and solar and activity during periods of normal undisturbed activity. Furthermore, these ANS responses are initiated at different times after the change in the various environmental factors and persist over different lengths of time. It should be noted that a common finding is that different individuals respond differently to changes in the same environmental variable^69^. In a separate analysis of the data presented here, collaborators at the Lithuanian University of Health Sciences developed a new measure for assessing sensitivity to magnetic field variations^92^. Although different participants had different responses at the individual level, the analysis presented in this paper concerns responses at the group level.

It is clear that a major driver of changes and disturbances in Earth’s magnetic field environment are the sun and solar wind^88,93^. Consistent with these findings, the solar wind speed was highly correlated with Kp, Ap and the PC(N) all of which reflect magnetic field disturbances. We also found that ULF power, which is related to magnetic field-line resonances, was positively correlated with solar wind speed, and indices of field disturbance was negatively correlated with cosmic ray counts which is consistent with the well-known inverse action of solar and geomagnetic activity and cosmic ray counts at the Earth’s surface^90^.

Regarding HRV responses, IBIs have an inverted relationship to heart rate where larger IBIs equated to a lower heart rate. Heart rate and IBIs are an ideal indicator of changes in the relative balance between parasympathetic and sympathetic activity and how the autonomic system responds and adapts to various types of stressors or challenges^40^. If an environmental variable is negatively correlated with IBIs, it indicates that heart rate increases with increases in that variable, which suggests a physiological stress reaction occurred. On the other hand, a positive correlation with IBIs indicates a lower heart rate. There were robust positive correlations between IBIs and Schumann Resonances and to a lesser degree with cosmic rays.

The positive correlation found between HF power and solar radio flux indicates an enhancement of parasympathetic nervous system activity during periods increased solar radio flux. This was of particular interest because a previous study with 1,643 participants in 51 countries found that the solar radio flux index was positively correlated with reduced fatigue, improved positive affect, and mental clarity while increases in solar wind speed had the opposite effects^94^. The potential beneficial effects of the solar radio flux was also observed in several studies that looked at death rates from various causes which found a strong and inverse relationship between the F10.7 and death rates^73,95^. The solar radio flux may be an important mediator of the anticipatory reactions observed by Tchijevsky which can occur several days before increases in the solar wind reach Earth and create magnetic disturbances. Of course, other sources of radiation such as X-ray, cosmic rays and UV emissions from the sun during coronal mass ejections are also likely aspects of the anticipatory reaction.

The strong and positive correlations between HF power and cosmic rays, as well as TP and VLF power, suggest a favorable response to increased cosmic rays, at least in a healthy population. The ANS response to increases in cosmic rays was immediate and continued throughout the forty-hour analysis window. It is clear from Fig. 3 that the largest ANS relationships were to cosmic rays, which supports the perspective of Stoupel, that cosmic rays are emerging as a principal factor of the environmental forces that affect human physiology^73^. The observations in this study, which suggests a positive reaction to cosmic rays, may seem at odds with the finding that increased cosmic rays, in conjunction with low geomagnetic activity, is positively associated with the timing of sudden cardiac deaths^72,95^ and strokes^73^. However, it may be that in only looking at death rates, which primarily reflect a sick and elderly population, that important aspects of how these environmental factors affect healthy populations could be overlooked. For example, in a large study of a younger population with potential inflammatory related problems who had serum C-reactive protein (CRP) tests taken, found a robust, inverse correlation between C-reactive protein levels and cosmic rays^96^. Interestingly, inflammation and higher levels of C-reactive protein have also been shown to be associated with lower levels of HRV, especially with a reduced VLF band^97,98^.

The other environmental variable that was strongly associated with increased the HF, LF and VLF power and TP of the HRV measures was Schumann resonance power (SRP). This was accompanied by the positive correlation to IBIs (lower heart rate), which was also significant during most of the analysis period. Also supporting a beneficial effect of enhanced SRP is a study that found reduced systolic, diastolic and mean arterial blood pressure during times of higher SRP^99^. Persinger and colleagues have conducted a number of studies showing that not only are the base rhythms of the brain similar, but real-time coherence between Schumann resonances and brainwaves can occur in participants globally, and that the intensity of the Schumann resonance is linearly related to the amount of coherence^83,100^. They also have proposed that information transfer can occur between human brains and the Schumann resonances^84^.

We have suggested that although the specific mechanisms are not yet clear, the energetic environmental factors discussed above either can directly or indirectly affect human psychophysiology and behaviors in different ways depending on the health status and maturity of the individuals^87^. This perspective is supported by the finding that increases in solar radio flux, cosmic rays and Schumann resonance power are all associated with increased HRV and parasympathetic activity. The ANS also appears to respond quickly to changes in cosmic rays, Schumann resonance power and the solar radio flux. These may well be some of the key drivers of Tchijevsky’s Index of Mass Human Excitability that clearly tracks the solar cycle^6^. On a larger societal scale, an increase in these energetic factors are associated with increased social unrest^3^, motivation^101^ and human flourishing^8^. The findings that both extremely high as well as extremely low values of geomagnetic activity are associated with the timing of increased death rates^72^ also suggests that our energetic environment affects people’s energy levels and that low activity or disturbances can act as triggers in sensitive and unhealthy populations and serves to motivate and facilitate human activity.

### Limitations

Although this type of study is correlational due to the independent measures of interest, it is a limitation. Additional merging of empirical results from a larger number of studies using different populations, designs, geographic locations and sampling increments, adds support to our findings. Although it is a truism that correlation does not imply causation, relationships generally must exist to be consistent with theories about causality.

Consideration of the logically possible causal relationships between correlated variables A and B allows only four alternatives. Either A and B are causally unrelated and their correlated variation is happenstance, or A causes B, or B causes A, or the correlated variations in A and B are jointly caused by some external factor. In the current analysis, the “happenstance” hypothesis is the one that is ruled out by the high significance level of the results. It is at least plausible that external electromagnetic effects contribute causally to changes in HRV, while it seems utterly absurd to posit the reverse causal relationship that HRV changes in a small population of individuals in one geographical region causes changes in solar and geomagnetic activity. The fourth case, that both phenomena are caused by yet another factor external to either, suffers from the lack of any theoretical candidate for such an external factor. As a result, the hypothesis that environmental electromagnetic effects cause changes in ANS dynamics appears to be the most plausible causal interpretation of the observations. Because no one study or form of evidence can be considered as definitive, support for causality can best be argued when various classes of evidence all converge on the same conclusion. We have therefore reviewed and documented multiple lines of evidence that have rigorously tested the hypothesis that daily ANS activity, as reflected by HRV, responds to changes in geomagnetic and solar activity at different times and persists over differencing periods of time. Moreover, the results presented are consistent with and extend previously published results. Unique to our findings is the observation that changes in the various ambient magnetic environment affect the human autonomic nervous system differently with specific temporal response patterns.

## Conclusions

Overall, this study strongly confirms that daily ANS activity, as reflected by HRV measures, responds to changes in geomagnetic and solar activity primarily during periods undisturbed by solar activity. Furthermore, these ANS responses are initiated at different times after the change in the various environmental factors and continue over different lengths of time. Solar wind was negatively correlated with IBIs indicating that heart rate increases with increases in solar wind that suggests a physiological stress reaction occurred. It appears that increased cosmic rays, solar radio flux, and Schumann resonance power are all associated with increased HRV and increased parasympathetic activity, and the ANS responds quickly to changes in these environmental factors. These may well be some of the key drivers of Tchijevsky’s Index of Mass Human Excitability that clearly tracks the solar cycle. These findings support the hypothesis that these energetic environmental factors act as energy sources that outplay in different ways depending on an individual’s health status and maturity level and capacity of self-regulation.

## Electronic supplementary material

Supplementary Information
