# Supplementary material for: Long-Term Study of Heart Rate Variability Responses to Changes in the Solar and Geomagnetic Environment
Source: Sci Rep. 2018 Feb 8;8:2663. doi: 10.1038/s41598-018-20932-x (PMC5805718; doi:10.1038/s41598-018-20932-x)
Supplement: Supplementary file 1 — Supplementary Information [file 41598_2018_20932_MOESM1_ESM.doc]

**Long-Term Study of Heart Rate Variability Responses to Changes in the Solar and Geomagnetic Environment**

**Supplementary Information**

Abdullah Alabdulgader,^1^ Rollin McCraty,^2^ Michael Atkinson^2^, York Dobyns^2^, Alfonsas Vainoras,^3^ Minvydas Ragulskis^4^ and *Viktor Stolc^5^

1 Prince Sultan Cardiac Center. Alhasa/Saudi Arabia

2 HeartMath Institute, Boulder Creek, CA

3 Lithuanian University of Health Sciences, Lithuania

4 Kaunas University of Technology, Kaunas, Lithuania

5 NASA Ames Research Center, Moffett Field, CA
